# Delivering into the Mounting Evidence of a Probable Link between Maternal Hypothyroidism and Breech Presentation at Term: What Do We Know until Now?

**DOI:** 10.3390/jcm10051120

**Published:** 2021-03-08

**Authors:** Panagiotis Peitsidis, Nikolaos Vrachnis

**Affiliations:** 1 Department of Obstetrics and Gynecology, Tzaneio Hospital Piraeus, National Public Health Organisation, 18536 Piraeus, Greece; 2 Department of Midwifery University of West Attica, 12243 Athens, Greece; 3 3rd Department of Obstetrics and Gynecology, National and Kapodistrian University of Athens Medical School, Attikon Hospital, 12462 Athens, Greece; nvrachnis@hotmail.com; 4 Vascular Biology, Molecular and Clinical Sciences Research Institute, St George’s University of London, London SW17, UK

Approximately 3–5% of all women at term have a breech baby. In this case, for the fetus, a planned cesarean section (CS) is considered a better choice than a planned vaginal birth, since, comparing the two modes, perinatal mortality is slightly reduced with CS [1,2,3,4]. However, CS for breech also presents some risks. The etiology of breech presentation has not been elucidated to date, with factors such as multiple pregnancy, multiparity, pelvic and uterine abnormalities, prematurity, placenta previa, fetal growth restriction, polyhydramnios, and umbilical cord and congenital fetal abnormalities accounting for only 15% of breech presentations [5].

In the last two decades, evidence has emerged showing that endocrinological disorders may cause decreased fetal movements due to pituitary pathology, with this possibly leading to breech presentation, meaning the inability of the thyroid gland to produce sufficient amounts of thyroid hormone. The prevalence of hypothyroidism in pregnancy is estimated to be 0.3–0.5% in the case of overt hypothyroidism and 2–3% in subclinical hypothyroidism (SCH). Moreover, an underactive thyroid is accompanied by high thyroid-stimulating hormone (TSH) levels, i.e., above 10 mU/L, with this condition being seven times more likely to occur in women than in men [6].

In the last decade, a large number of study groups have examined a potential association between hypothyroidism and breech presentation, with this arousing much research interest. Specifically, a possible association not only of overt hypothyroidism but also of SCH has been investigated, and, in 2004, the first study was published exploring the role of low levels of maternal thyroxin as a risk factor for breech presentation [7]. The authors reported that, in a small cohort of 204 pregnant women, 12 of them had breech presentation, with 10 (85%) of these women having free thyroid hormone (fT4) levels <10 percentile at 12 weeks of gestation (OR 4.7, 95% CI 1.1–19). However, at 24 and 32 weeks of gestation, no link between fT4 levels and fetal presentation was observed; it was thus concluded that neither hypothyroidism nor SCH was related to breech presentation. Describing their findings, the authors hypothesized that the frequency and intensity of fetal movement is essential to establishing cephalic presentation; limited movement, in contrast, leads to the development of a shorter umbilical cord, which, in turn, limits the baby’s movement, resulting in breech presentation. In addition, it is highlighted that because, as mentioned, increased maternal TSH reflects suboptimal maternal thyroid function, this will affect T4 transfer from the mother to the fetus, ultimately leading to impaired fetal neuromotor outcome.

This report was followed 5 years later by several more studies on a possible association between hypothyroidism and breech presentation. In 2010, Kooistra et al. [8], in a large cohort of more than 1000 women, reported their findings in 189 women with breech presentation. They assessed three thyroid parameters, namely, fT4, TSH, and thyroid peroxidase antibody (TPO-Ab), and found that women with high TSH towards the end of pregnancy are at risk for breech presentation. In addition to endorsing the previously proposed explanation of impaired fetal movement, the authors put forward the hypothesis that TSH may directly act to modulate uterine contractility, thus limiting further fetal movement.

In the same year, the above study group published another study [9] of 58 women who had delivered with breech presentation; they evaluated the levels of TSH at 36 weeks compared to those in women who had delivered with cephalic presentation, this time using a TSH greater than 2.5 mIU/L as the cut-off value. Their results showed that women whose TSH levels are above 2.5 mIU/L at term are at increased risk for breech presentation. However, this is not the case if TSH is abnormal at 12 and 24 weeks gestation.

Again in 2010, another study was published in Canada with 179 women with infants delivered in breech presentation; the outcomes of this study conflicted with those of the previous study [10]. When serum thyroid hormone levels were examined at 15–16 weeks of gestation, no statistical association was found between TSH levels at 15–16 weeks of gestation and the breech gestation at >37 weeks. Although this study population was larger compared to those of previous studies, the authors reported several limitations.

In 2013, a large retrospective US cohort study by Mannisto et al. examined the electronic medical notes of 223,512 singleton pregnancies [11]. The authors’ findings showed that iatrogenic hypothyroidism alone (i.e., hypothyroidism due to thyroid surgery or ablation) was associated with a 2.1% higher incidence of breech presentation (OR 2.09, 99% CI 1.07–4.07), while primary hypothyroidism was not associated with breech presentation (OR 1.10, 99% CI 0.89–1.35). In 2016, a cohort study from Amsterdam was published including 3347 pregnant women, demonstrating that increased TSH and decreased FT4 during the second trimester of pregnancy are linked to a higher risk for term breech presentation [12]. On the other hand, it was unclear whether there was any association of abnormal thyroid parameters in the first or third trimester. This was the first study demonstrating a link between second-trimester increased TSH and breech presentation, as the previous studies had only shown an association of hypothyroidism at term. 

In 2017, Furnica et al. examined first-trimester thyroid hormone levels in 783 women with singleton low-risk pregnancies [13]. Defining hypothyroxinemia as fT4 levels below the 5th centile, the researchers found that hypothyroxinemia in the first trimester was associated with a 6.5 fold increase in breech presentation at term. 

Finally, the most recently published study (2020) is the Finnish cross-sectional study analyzing data from more 737,788 singleton births over a decade, which showed a clear association between hypothyroidism and breech presentation in term pregnancies (OR 1.68, 95% CI 1.41–2.01) [5]. This association was clearly demonstrated only when hypothyroidism was diagnosed after 37 weeks of gestation and not earlier, even when the period between 24 and 37 weeks was split into three groups (24–27 weeks, 28–32 weeks, and 33–37 weeks). The main strength of this study is that the analysis was based on a large nationwide population with homogenous medical treatment. On the other hand, limitations of the study include the retrospective design and the restriction of variables due to data availability.

All the above-mentioned studies provide strong evidence of an association between breech presentation at term and hypothyroidism. However, among these, while there are reports clearly pointing to a possible interaction, there are few containing conflicting data or demonstrating that there is no statistically significant correlation. It must nonetheless be kept in mind that the existing studies have limitations in their design and data collection, thus raising some concerns as to the strengths of their outcomes. To resolve this issue, randomized controlled trials and prospective trials are warranted which, would provide a higher level of evidence.

Another point that needs further clarification is the underlying causes of the presumed association between hypothyroidism and breech presentation, with there being hypotheses but no clear explanation as to why this happens to date. Moreover, while most existing studies show a connection between hypothyroidism at the end of pregnancy and breech presentation, the possible effect of hypothyroidism early in pregnancy on breech presentation, leading to a breech presentation at term, remains unclear. More studies of high quality will, in future, need to be designed so that safer conclusions may be reached. The difficulties and ethical concerns that are raised with randomized trials on pregnant women must, however, be kept firmly in mind. 

What must be particularly stressed once again is the necessity for optimal thyroid disease management of women during any pregnancy that is clearly associated with potential for poor fetal and maternal outcome. As concerns the implication of maternal hypothyroidism in breech presentation, and thus a possible adverse perinatal outcome, it is as yet uncertain whether breech presentation and delivery may be attributed solely to maternal thyroid dysfunction or to a combination of factors. Further research on this vital issue is, therefore, required to establish the specific contribution of hypothyroidism to the perinatal outcome.
